# Year‐Round Evaluation of the Conservation Potential of Seed‐Rich Field Margins Under Agri‐Environmental Schemes for Farmland Birds, European Hares, and Common Hamsters

**DOI:** 10.1002/ece3.72492

**Published:** 2025-11-18

**Authors:** Martin Šálek, Ondřej Boháč, Jan Cukor, Peter Samaš

**Affiliations:** ^1^ Czech Academy of Sciences Institute of Vertebrate Biology Brno Czech Republic; ^2^ Faculty of Environmental Sciences Czech University of Life Sciences Prague Prague Czech Republic; ^3^ Forestry and Game Management Research Institute Jíloviště Czech Republic; ^4^ Czech Society for Ornithology Prague Czech Republic; ^5^ Faculty of Forestry and Wood Sciences Czech University of Life Sciences Prague Prague Czech Republic

**Keywords:** common agricultural policy, common hamsters, conservation measures, farmland eco‐schemes, full annual cycle, habitat quality, mammals, seed‐rich strips

## Abstract

Field margins on arable land, such as seed‐rich strips (SRS), are an important and widely implemented conservation measure under agri‐environment schemes. They are intended to contribute to the provision of scarce food resources and shelter for various farmland species. Although SRS are primarily designed to provide critical resources for wildlife during the winter, they may also contribute to biodiversity conservation throughout the year and across the full annual cycle of farmland species. However, empirical evaluation of their conservation potential over the annual cycle and across multiple taxa is missing. Therefore, in this study, we conducted a year‐round assessment of the conservation potential of SRS for several avian (farmland birds and seed‐eating species in particular) and mammalian (European hares and common hamsters) farmland species in arable‐dominated agricultural landscapes. Moreover, we investigated the role of the habitat context (i.e., SRS placed along farmland hedges or inside crop fields) of SRS on species abundance and diversity. Our results showed that during the entire year, SRS significantly increased farmland bird abundance and species diversity (including seed‐eating species), as well as European hare and common hamster abundance, compared to the controls (i.e., conventionally managed arable land). The SRS utilization varied seasonally, with bird abundance being significantly higher from late summer through winter, while European hare abundance increased significantly only in the autumn and winter months. Notably, the most substantial differences in farmland bird diversity between the SRS and control areas were observed from August to November, whereas the greatest disparities in bird abundance occurred during the winter months, particularly from December to February. The habitat context of the plots (both SRS and controls) generally did not affect the abundance nor species diversity of farmland birds. However, the abundance and species diversity of seed‐eating birds were higher in plots located along hedges. The opposite pattern was found for the European hare. We conclude that implementing SRS could significantly help in the conservation of avian and mammalian species in arable farmlands. This is true not only in the autumn and winter, when the dense and structurally complex vegetation in SRS offers abundant nutrient‐rich seeds and cover, but also in the summer, when SRS provide invertebrates for a variety of farmland species.

## Graphical abstract

Field margins on arable land, such as seed‐rich strips (SRS), are an important and widely implemented conservation measure under agri‐environment schemes. Although SRS are primarily designed to provide critical resources for wildlife during the winter, they may also contribute to biodiversity conservation throughout the year and across the full annual cycle of farmland species. However, empirical evaluation of their conservation potential over the year and multiple taxa is missing. We found that implementing SRS could significantly help in protecting avian and mammalian species in arable farmlands—not only in the autumn and winter, when the dense and structurally complex vegetation in SRS offers abundant nutrient‐rich seeds and cover, but also during the summer when SRS provide invertebrates for a variety of farmland bird species.

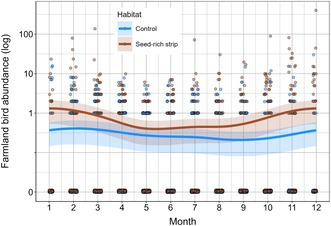

## 1 Introduction

The onset of intensive and large‐scale agricultural practices, especially since the 1950s, led to the rapid disappearance of traditional biodiversity‐rich farmlands created and maintained for millennia by small‐scale and extensive farming (Robinson and Sutherland 2002; Benton et al. 2003). Intensively managed farmlands are currently dominated by structurally simple and homogeneous landscape patterns with low crop diversity and minimal representation of (semi)natural and other extensively used habitats (Benton et al. 2003; Stoate et al. 2001, 2009), which has inevitably resulted in degraded biodiversity and related ecosystem services across large spatial scales (Donald et al. 2001; Tilman et al. 2001; Green et al. 2005; Tscharntke et al. 2005; Emmerson et al. 2016; Šálek et al. 2021, 2026). Halting biodiversity loss became one of the highest priorities in European nature conservation (EC 2020), reflected in the implementation of several conservation measures, including agri‐environment schemes (AES) under the Common Agricultural Policy (CAP).

The AES are a basic instrument of CAP that aims to promote ecologically friendly farming and enhance the representation of biodiversity‐rich habitats on various farmed habitats (Batáry et al. 2015; Hodge et al. 2015). Although their benefits for farmland biodiversity conservation were frequently disputed in the past (Kleijn et al. 2001, 2011; Pe'er et al. 2019), some AES conservation measures may have a positive effect on biodiversity, particularly within cropland‐dominated farming systems (Batáry et al. 2011, 2015; Staggenborg and Anthes 2022). For example, set‐asides, fallow land, organic farming, and field margins are regarded as effective measures for the promotion of farmland biodiversity on arable land (Van Buskirk and Willi 2004; Bengtsson et al. 2005; Vickery et al. 2009; Haaland et al. 2011; Pe'er et al. 2019; Sanz‐Pérez et al. 2021; Staggenborg and Anthes 2022). Farmers often use various types of field margins to protect the environment, among which flower‐rich and seed‐rich strips are the most popular AES (Marshall and Moonen 2002; Vickery et al. 2009; Haaland et al. 2011; Dicks et al. 2013; Tyllianakis et al. 2025). Though they both may be beneficial for a high diversity of farmland species, they are established to support different wildlife taxa (Haaland et al. 2011; Dicks et al. 2013). Flower‐rich strips may enrich the insect biodiversity and ecosystem services through pollination or conservation biocontrol (Haaland et al. 2011). Seed‐rich strips (i.e., sown strips with seed‐rich mixtures; hereafter referred to as SRS), on the other hand, are implemented to support (and reverse negative population trends of) avian and mammalian farmland species through the provision of vital—and limiting—seed food and cover resources in the agricultural landscape (Marshall and Moonen 2002; Dicks et al. 2020; Šálek, Bažant, et al. 2022).

The principal conservation benefits of SRS are expected to be most pronounced in the winter (Dicks et al. 2013) when many farmland birds (especially seed‐eating species) and mammals rely on these seed‐rich habitats for food, while high and dense vegetation provides them with crucial cover and shelter against unfavorable weather conditions and predators (Dicks et al. 2020; Šálek, Bažant, et al. 2022). Especially in Northern and Central Europe, winter is a critical period in the life cycle of mammals and sedentary or short‐distance migratory birds (Wilson et al. 2009) as food resources are generally limited, which may result in reduced survival (Siriwardena et al. 2000; Hole et al. 2002). In particular, previous evidence has shown that many farmland taxa/species, including farmland birds, small mammals, and European hares, selected SRS over the surrounding farmland during the winter (Marshall and Moonen 2002; Dicks et al. 2020; Šálek, Bažant, et al. 2022). However, SRS may also support multiple farmland species through the provision of essential food and shelter resources in different phases of their annual cycles. Moreover, the conservation potential of SRS can also vary depending on other factors, such as agricultural management (i.e., establishment, cutting, harvesting, or herbicide use), seed mixtures, vegetation structure, and spatial distribution (habitat context) in the landscape (Marshall and Moonen 2002; Vickery et al. 2009; Tscharntke et al. 2011; Dicks et al. 2013). In particular, the spatial distribution of SRS (e.g., SRS placed along the field boundaries/hedges or inside the crop field blocks) creates contrasting and highly dynamic vegetation conditions for different farmland species throughout the year.

Similar to other components of farmland biodiversity, many vertebrates—habitat specialists in particular—have undergone rapid population decline across different European agroecosystems (Robinson and Sutherland 2002; Green et al. 2005; Smith et al. 2005; Šálek et al. 2013). One of the best‐known and documented population reductions is that of farmland birds, whose population has decreased by 61% since 1980 (PECBM 2023). A similar negative population trend is known for mammalian farmland specialists, such as the European hare *Lepus europaeus* or common hamster *Cricetus cricetus* (Smith et al. 2005; Surov et al. 2016). Once abundant and widely distributed species experienced considerable population reduction in the last few decades, which resulted in several local extinctions and fragmentation of their former distribution ranges (Edwards et al. 2000; Smith et al. 2005; Surov et al. 2016; Pavliska et al. 2018). The factors contributing to the population decline may vary among species and taxa. However, these negative population trends are generally linked to the loss of crucial habitat and foraging opportunities, closely linked with the intensification of agricultural practices, and reduced landscape heterogeneity and extensively used biodiversity‐rich habitats (Benton et al. 2003; Tscharntke et al. 2005; Šálek, Kalinová, and Reif 2022; Ševčík et al. 2025). Hence, creating resource‐rich field margins (along with other AES conservation measures) on arable land may profoundly enhance habitat heterogeneity and support the declining farmland biodiversity.

Previous research on the SRS conservation potential for farmland vertebrates has focused only on a few species (Perkins et al. 2002; Douglas et al. 2009) and only explored their conservation potential during a single season—specifically in winter (Henderson et al. 2007; Šálek, Bažant, et al. 2022)—but we lack conservation evidence on how SRS may help a wide range of farmland species throughout their annual cycles, including migration, breeding, post‐breeding, and dispersal (see also Šálek et al. 2025). Having this knowledge could significantly improve our conservation efforts. Therefore, in this study, we studied the year‐round conservation potential of SRS for avian and mammalian farmland species in cropland‐dominated farming systems in Central Europe. More specifically, we assessed seasonal changes in abundance and species diversity of farmland birds, European hares, and common hamsters (as representatives of mammals) in SRS and compared the acquired data with arable habitats in the surrounding farmland. We also explored how the landscape context (i.e., SRS located along farmland hedges or inside crop fields) affects the abundance and species diversity of the studied taxa/species. We hypothesized that SRS would support a higher abundance and species diversity of farmland wildlife species throughout the entire year compared to the surrounding farmland. Additionally, based on previous evidence from similar seed‐rich and wildflower habitats and eco‐schemes (e.g., Vickery et al. 2009; Haaland et al. 2011; Tscharntke et al. 2011; Dicks et al. 2013; Rieger et al. 2022), we predicted significant seasonal variations in abundance and species diversity of surveyed taxa/species within the SRS. These variations reflect individual species' demands throughout the year but also resource availability (habitat quality) of SRS (reflecting seasonal changes in the farming management, vegetation composition, and succession). In particular, we expected that SRS would have the highest conservation potential in the autumn and winter (i.e., migration and wintering periods), particularly for seed‐eating birds and European hares. During these seasons, SRS should provide ample food (seeds) and shelter resources. However, the quality and quantity of food and shelter resources can decrease in the second half of winter (due to depleted seed availability and vegetation cover), followed by further collapse in April when SRS are plowed and re‐sown. Furthermore, the subsequent succession of SRS vegetation may support a higher abundance and diversity of different wildlife species from the late summer (i.e., late breeding and post‐breeding periods) because their complex vegetation cover provides shelter and greater availability of invertebrates. Finally, we expected similar abundance and species diversity of farmland birds in SRS with different habitat contexts, due to their species‐specific affinity to various habitat structures. However, some species, including seed‐eating birds, may benefit from extra resources available from nearby farmland hedges.

## 2 Material and Methods

### 2.1 Study Area

This study was performed in arable‐dominated lowland farmland in Central Moravia, Czech Republic, Central Europe (altitude range: 230–270 m a.s.l., GPS centroid: 49.64° N, 17.11° E). The structure of the agricultural and rural landscape in the Czech Republic was heavily influenced by state‐controlled land consolidation programs initiated in the 1950s, resulting in large‐scale homogenization of landscape patterns. Currently, Czech arable fields are among the largest in Europe (Šálek et al. 2018; Clough et al. 2020). The farmland landscape is characterized by large arable fields, predominantly used for the cultivation of cereals, rapeseed, maize, and sugar beets. These fields are interspersed with a limited array of non‐farmed habitats, especially farmland hedges, small woodlots, shrubby patches, and ditches.

In our study area, seed‐rich strips under agri‐environmental schemes consisted of linear strips on arable land (either crop field edges or within the fields) that were between 338 and 989 m (average = 590 m) in length and 12–24 m (average = 19.6 m) wide. The minimal distance between two adjacent SRS was 1022 m. There are several conditions for planting SRS in the Czech Republic: (i) SRS must be cultivated for at least five consecutive years, (ii) application of pesticides and fertilizers is prohibited, (iii) SRS are established by mid‐June and maintained without agricultural management until the end of March, when they are plowed and re‐sown, and (iv) SRS are sown with special seed mixtures (Ministerstvo zemědělství 2016). The seed mixtures consist of mandatory plant species: spring cereals ( *Avena sativa* , *Triticum aestivum* , or *Hordeum vulgare* ), millet (*Panicum miliaceum*), wild cabbage (*Brassica oleracea*), and buckwheat (*Fagopyrum esculentum*). Optional plant species include sunflower (*Helianthus annuus*), canary grass (*Phalaris canariensis*), lacy phacelia (*Phacelia tanacetifolia*), blue curls (*Phacelia congesta*), flax (*Linum usitatissimum*), white lupin (*Lupinus albus*), *sorghum* (*Sorghum bicolor*), foxtail millet (*Setaria italica*), and legumes. The complete enumeration of mandatory and optional plant species utilized for the establishment of SRS and their minimum quantity in the mixture (kg/ha) is shown in Table S1. Moreover, SRS are frequently colonized by weeds from the surrounding fields and non‐cropped vegetation (e.g., *Cirsium arvense* , *Chenopodium album* , *Helianthus tuberosus* , and *Papaver rhoeas* , among many others).

### 2.2 Study Design and Biodiversity Monitoring

The study protocol was based on a paired design—one study plot comprised of SRS and a corresponding control site on arable land (hereafter referred to as controls; see also Šálek and Żmihorski 2018; Šálek et al. 2020; Šálek, Kalinová, and Reif 2022). First, the locations of studied SRS were selected based on their locations from previous years (data provided by the Ministry of Agriculture of the Czech Republic). Second, the location (and the length) of each SRS was determined using GIS software (QGIS 2012) and detailed satellite maps (Mapy.cz). Control transects were located in adjacent farmland within 500 m from paired SRS, had the same strip length, and were situated at the same or neighboring field blocks to mitigate potentially confounding variables, such as environmental and climatic conditions, land‐use composition, or agricultural management (see also Šálek, Bažant, et al. 2022). All study plots were located more than 200 m away from the main roads to avoid a noisy acoustic background. In total, we surveyed ten study plots (i.e., ten SRS and ten controls), five of which were situated along farmland hedges (linear strips of shrubby and tree vegetation within farmland) and five within the crop field interior (at least 100 m from the crop field edge).

The selected farmland vertebrates were surveyed using the linear transect method, recording all observed individuals/burrows within designated buffers during slow walks (< 2 km/h). In particular, we recorded the abundance and species diversity of farmland birds and the abundance of European hares in a 100 m buffer (i.e., 50 m on each side of the survey transects). We also counted the number of European hamsters' burrows in a 10 m buffer (i.e., 5 m on each side of the survey transects, seen from the top while walking along the transect). We selected a narrower buffer for monitoring common hamsters due to dense and structural vegetation that makes it difficult to detect hamsters' burrows, especially in the growing season. Birds flying high over study plots without landing during the survey (e.g., geese, gulls, migrating raptors) were excluded from the counts, except for typical aerial foragers such as barn swallows ( *Hirundo rustica* ). This exclusion was based on the assumption that they were not directly associated with the studied habitats (see also Šálek, Kučera, et al. 2015; Šálek et al. 2018).

The research was conducted throughout the entire year (from February 2023 to January 2024), with each study plot surveyed once per month, approximately mid‐month (±4–5 days), in the morning (6:00–12 CEST). While the selected time for the biodiversity survey is suitable for birds, due to their peak vocal and foraging activity (Catchpole and Slater 2008), European hares are primarily nocturnal animals with peak activity at dusk and dawn (Schai‐Braun et al. 2012). Moreover, European hares tend to select denser vegetation during the day and move to open areas at night. This leads to different habitats being selected in active and inactive periods (Schai‐Braun et al. 2012; Mayer et al. 2018). Therefore, our sampling is not suitable for the evaluation of the species' true abundance and rather reflects their daytime (resting) preferences. The monitoring was performed under suitable meteorological conditions (i.e., no precipitation, snow, fog, or strong wind) by a single highly experienced surveyor (OB) to avoid bias in animal detection and identification by more field surveyors. Finally, SRS and control sites were always surveyed on the same day and in random order.

### 2.3 Statistical Analyses

To explore the conservation potential of SRS on farmland biodiversity, we only considered bird species that frequently use various farmland habitats for breeding or foraging year‐round, and therefore, we excluded typical forest, urban, and wetland species (see also Šálek et al. 2018, 2025). Moreover, for further analyses, we also selected a subset of seed‐eating birds (based on classification in Reif, Vermouzek, et al. 2010; Reif, Telenský, et al. 2010)—the primary target group whose overwintering survival can be directly increased through the SRS (Ministerstvo zemědělství 2016).

All statistical analyses were conducted using R version 4.3.2 (R Core Team 2023). For all abundance models, both observed (presence) and true zero (absence) counts were explicitly included in the dataset, ensuring that instances of no detection for a given species, plot, and month were treated as zeros in the analysis. Exceptions were made for combinations where the occurrence was biologically implausible (i.e., migratory species outside of the relevant season), which were omitted. To examine the year‐round dynamics of abundance and species diversity of farmland animals, we employed Generalized Additive Models (GAMs) using the function gam in the mgcv package (Wood 2017). We examined the effects of the time of the year (continuous circular variable; month), plot habitat (categorical; SRS and controls), and habitat context (categorical; within the field and along hedges). For the abundance models, we explicitly included both main effects (plot habitat and habitat context) as well as their interaction (plot habitat × habitat context) in our initial model specifications. The time period modeled at monthly resolution allowed us to (i) model seasonal patterns at a finer scale compared to broader categorical divisions (e.g., four seasons) and (ii) implement it as a continuous variable, which required fewer degrees of freedom and maintained higher statistical power. The time period was modeled using a cyclic cubic spline to account for its circular nature and was included in the interaction with the plot habitat to explore between‐category variation. We accounted for varying lengths of sampled study plots by including the offset of transect length (continuous; meters). We applied a negative binomial distribution with a log link in the set of models testing dynamics of abundance (response variable count of animals). In this set of models, we used the same structure to test datasets for farmland birds, seed‐eating birds and European hares only. Random intercept included species ID (except for the single‐species subset with the European hares and common hamster) to account for repeated species observations within each unique month–plot habitat–habitat context combination. Another random intercept included plot ID to account for repeated monthly sampling in each plot. To test for the interactive effect of plot habitat and habitat context, we included both main effects and their interaction in the initial model. However, to make the models simpler and the main effects easier to understand, we systematically removed this interaction term whenever it was clearly non‐significant, and we retained only the terms supported by the data or hypothesized as essential to the study questions. Our approach was primarily hypothesis‐driven: all main effects (time, plot habitat, and habitat context) were specified a priori based on our study design and ecological hypotheses about seasonal and spatial variation in farmland biodiversity. We, therefore, fitted full models without model selection to avoid excluding potentially meaningful ecological effects, even if they were not statistically significant in our specific dataset. Due to insufficient data for the common hamster, we excluded the effect of time period from the GAM model. To test the above effects on alpha species diversity in farmland and seed‐eating species, quantified by the Shannon index, we employed a comparable statistical framework. The only modification involved utilizing a Tweedie distribution with a logarithmic link function particularly suited for handling zero‐inflated continuous positive data, while omitting the needless random effect of species ID. Residuals from all models were examined using the package DHARMa v. 0.4.6, which allows diagnostics of non‐normally distributed models using simulated residuals (Hartig 2022). Post hoc comparisons of model‐adjusted marginal means among plot habitats and months were performed using the package emmeans v. 1.10.1 (Lenth 2024).

## 3 Results

In total, we recorded 3576 individuals of 46 farmland bird species, including 15 seed‐eating bird species (*n* = 2300), 341 individuals of European hare and 80 burrows of common hamster. A list of farmland species recorded in SRS and corresponding controls, their diet, sum of individuals, mean abundance and SD for both habitats (SRS and controls) is shown in Table S2.

### 3.1 SRS Use Over an Entire Year

**FIGURE 1:**
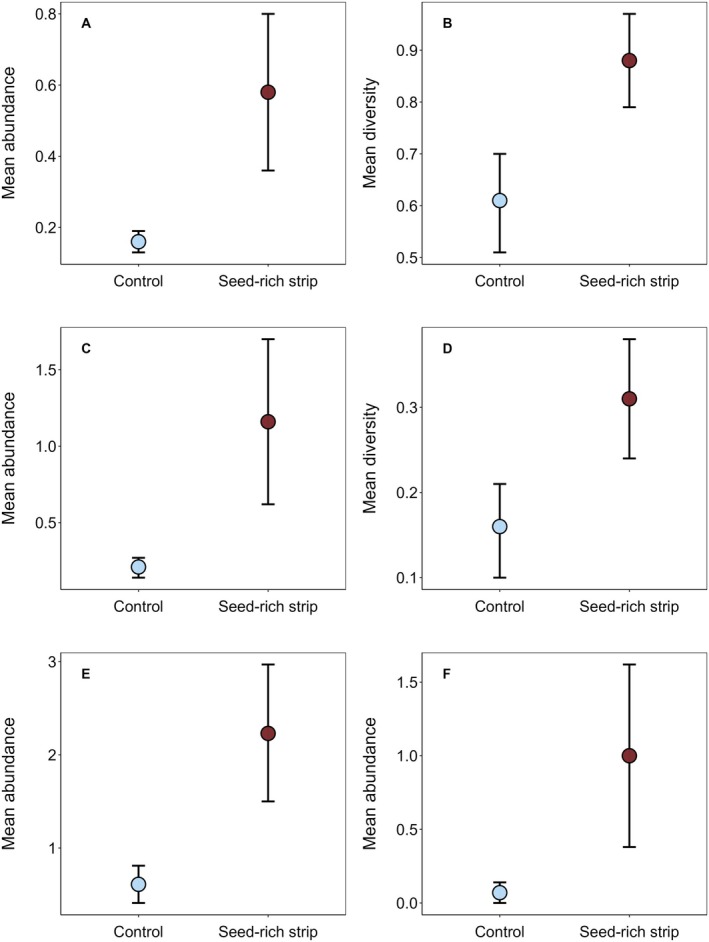
Comparison of seed‐rich strips versus control plots for the studied ecological metrics of (A) Abundance of farmland birds, (B) Alpha diversity of farmland birds, (C) Abundance of seed‐eating birds, (D) Alpha diversity of seed‐eating birds, (E) Abundance of European hare, and (F) Abundance of common hamster. All data represent observed means over the entire year with error bars indicating 95% confidence intervals. Means and confidence intervals were calculated including zero values (absences and undetected species).

Farmland bird abundance recorded on the study transects was significantly higher in SRS compared to control plot habitats (GAM: estimate = 0.84 ± 0.11, *χ* ^2^ = 7.73, *p* < 0.001; Figure 1; Table S3). The Shannon diversity in farmland birds was also significantly higher in SRS (GAM: estimate = 0.39 ± 0.09, *χ* ^2^ = 4.47, *p* < 0.001; Figure 1; Table S4). In the seed‐eating birds, the same pattern was found for both the abundance (GAM: estimate = 1.46 ± 0.18, *χ* ^2^ = 8.08, *p* < 0.001; Figure 1; Table S3) and Shannon diversity (GAM: estimate = 0.86 ± 0.25, *χ* ^2^ = 3.42, *p* < 0.001; Figure 1; Table S4). Mammals followed the same pattern as farmland birds, with significantly higher abundances in SRS observed in both European hare (GAM: estimate = 0.86 ± 0.22, *χ* ^2^ = 3.86, *p* < 0.001; Figure 1; Table S3) and common hamster (GAM: estimate = 2.45 ± 0.68, *χ* ^2^ = 3.61, *p* < 0.001; Figure 1; Table S5).

The habitat context (i.e., within the field or along hedges) did not have a significant effect on either the abundance or Shannon diversity in either SRS or control habitat plots (interaction of the plot habitat and habitat context: all *p* > 0.05). However, for seed‐eating birds, both the abundance (estimate = 1.17 ± 0.41, *χ* ^2^ = 2.87, *p* = 0.004; Table S3) and Shannon diversity (estimate = 1.122 ± 0.29, *χ* ^2^ = 4.19, *p* < 0.001; Table S4) were higher in plots (both control and SRS) located along hedges. Interestingly, the European hare abundance tended to decrease in plots along hedges (estimate = −0.87 ± 0.38, *χ* ^2^ = −2.30, *p* = 0.02; Table S3).

### 3.2 Seasonal Variation of SRS Use

**FIGURE 2:**
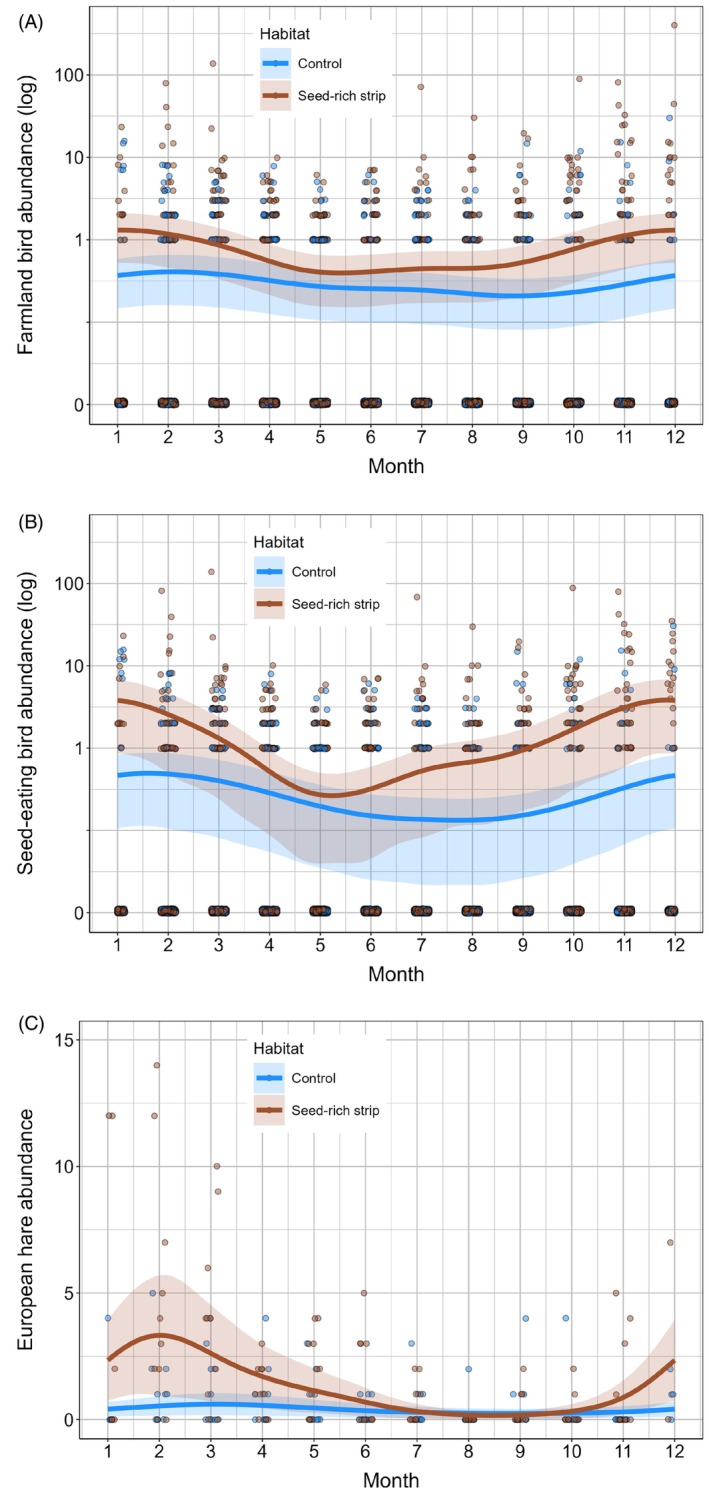
Seasonal variation in animal abundance (log scale) of seed‐rich strips versus control plots for (A) farmland bird species, (B) seed‐eating bird species, and (C) European hare. Model‐predicted trends (solid lines) with 95% confidence intervals (shaded areas) are shown alongside raw data points (circles).

The seasonal variation in the abundance of both farmland and seed‐eating birds was more pronounced in SRS, with higher SRS abundances observed especially during the winter months, particularly from December to February (Figure 2A,B; Table 1). The difference in bird abundances between SRS and control sites was less pronounced from April to June. A similar seasonal pattern was also observed for the European hare (Figure 2C).

**TABLE 1:** Post hoc comparisons of differences in farmland birds (left columns), seed‐eating birds (middle columns) and European hare (right columns) abundance between control and seed‐rich strip plots. Bold values indicate statistically significant differences (*p* < 0.05). Negative estimates indicate higher abundance in seed‐rich strip plots compared to control plots.

Month	Farmland birds			Seed‐eating birds			European hares		
	Estimate	SE	*p*	Estimate	SE	*p*	Estimate	SE	*p*
1	−1.274	0.203	**< 0.001**	−2.099	0.309	**< 0.001**	−1.741	0.345	**< 0.001**
2	−1.056	0.214	**< 0.001**	−1.666	0.335	**< 0.001**	−1.821	0.363	**< 0.001**
3	−0.808	0.215	**< 0.001**	−1.189	0.352	**0.001**	−1.467	0.375	**< 0.001**
4	−0.541	0.212	**0.011**	−0.6	0.368	0.103	−1.109	0.385	**0.004**
5	−0.399	0.216	0.065	−0.322	0.39	0.409	−0.92	0.406	**0.024**
6	−0.476	0.221	**0.032**	−0.757	0.4	0.059	−0.673	0.438	0.126
7	−0.597	0.222	**0.007**	−1.356	0.395	**0.001**	−0.138	0.479	0.773
8	−0.719	0.219	**0.001**	−1.634	0.384	**< 0.001**	0.311	0.509	0.542
9	−0.933	0.218	**< 0.001**	−1.823	0.372	**< 0.001**	0.276	0.505	0.585
10	−1.207	0.222	**< 0.001**	−2.081	0.363	**< 0.001**	−0.258	0.467	0.581
11	−1.361	0.218	**< 0.001**	−2.243	0.346	**< 0.001**	−1.058	0.406	**0.01**
12	−1.274	0.203	**< 0.001**	−2.099	0.309	**< 0.001**	−1.741	0.345	**< 0.001**

**FIGURE 3:**
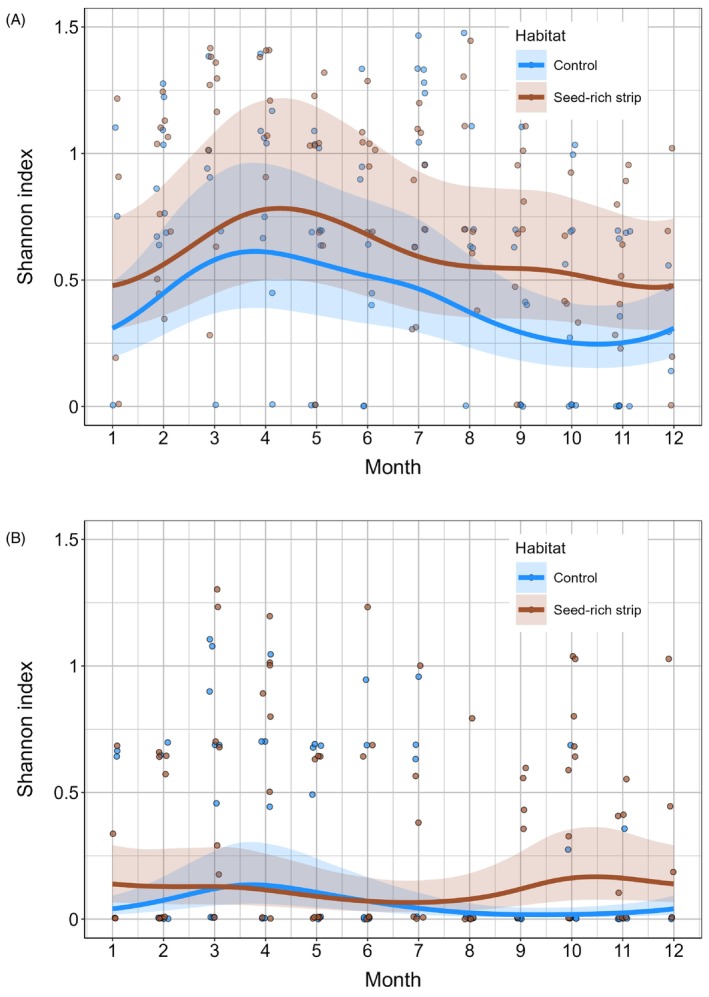
Seasonal variation in alpha diversity (Shannon index) in seed‐rich strips versus control plots for (A) farmland and (B) seed‐eating bird species. Model‐predicted trends (solid lines) with 95% confidence intervals (shaded areas) are shown alongside raw data points (circles).

The Shannon diversity for both farmland and seed‐eating birds was also higher in SRS compared to controls with the difference being most pronounced in the autumn and winter (Figure 3, Table 2).

**TABLE 2:** Post hoc comparisons of differences in farmland (left columns) and seed‐eating (right columns) bird alpha diversity (Shannon index) between control and seed‐rich strip plots. Bold values indicate statistically significant differences (*p* < 0.05). Negative estimates indicate higher abundance in seed‐rich strip plots compared to control plots.

Month	Farmland birds			Seed‐eating birds		
	Estimate	SE	*p*	Estimate	SE	*p*
1	−0.434	0.161	**0.007**	−1.229	0.413	**0.003**
2	−0.226	0.159	0.158	−0.546	0.42	0.196
3	−0.182	0.156	0.245	−0.066	0.43	0.877
4	−0.243	0.153	0.113	0.148	0.435	0.735
5	−0.29	0.155	0.063	0.16	0.45	0.722
6	−0.272	0.162	0.094	0.001	0.472	0.998
7	−0.243	0.169	0.152	−0.426	0.49	0.386
8	−0.396	0.175	**0.025**	−1.166	0.499	**0.02**
9	−0.621	0.182	**0.001**	−1.891	0.499	**< 0.001**
10	−0.731	0.184	**< 0.001**	−2.175	0.485	**< 0.001**
11	−0.651	0.176	**< 0.001**	−1.88	0.451	**< 0.001**
12	−0.434	0.161	**0.007**	−1.229	0.413	**0.003**

## 4 Discussion

In our study, exploring SRS conservation performance throughout the year and during the entire annual cycle of studied species, we demonstrated that SRS may be an effective tool for supporting farmland birds, European hares, and common hamsters. However, analysis of seasonal and species‐specific changes in SRS utilization also indicated that their conservation potential for individual taxa/species may vary substantially throughout the year.

In concordance with previous conservation evidence on the positive effect of various types of field margins on farmland biodiversity (Vickery et al. 2009; Dicks et al. 2013; Šálek, Bažant, et al. 2022), we found that SRS hosts a greater abundance and/or species diversity of farmland birds (including seed‐eating species), European hares, and common hamsters compared to controls (i.e., conventionally managed arable land). The importance of SRS and other seed‐rich habitats (e.g., set‐asides, bird cover crops) for supporting granivorous farmland birds was previously highlighted across various farmland types (Boatman et al. 2003; Perkins et al. 2008; Vickery et al. 2009; Dicks et al. 2013; Šálek, Bažant, et al. 2022; Hološková et al. 2025). Field margins and wildflower strips are valuable high‐quality habitats for European hares (Petrovan et al. 2013; Meichtry‐Stier et al. 2014; Sliwinski et al. 2019; Šálek, Bažant, et al. 2022), common hamsters (Fischer and Wagner 2016), and also for other vertebrates and insects inhabiting the agricultural landscape (e.g., Arlettaz et al. 2010; Hof and Bright 2010; Zellweger‐Fischer et al. 2011; Ouvrard and Jacquemart 2018; Peter et al. 2021).

SRS and other field margins support higher biodiversity due to the provision of essential nutrient‐rich resources for many avian and mammalian species (Boatman et al. 2003; Vickery et al. 2009; Dicks et al. 2013). Above all, they offer a high abundance and diversity of seeds and grains, whether from seeding mixtures or arable and ruderal weeds. Plant foods are considered a pivotal dietary source for birds (especially for a wide range of seed‐eating bird species) and mammals (including European hares and common hamsters), during the non‐breeding periods (Wilson et al. 1999). Moreover, SRS also hosts a wide array and quantity of arthropod species (Vickery et al. 2009; Haaland et al. 2011; Hološková et al. 2025), a crucial prey for a high diversity of birds (chicks and adults) in the breeding and post‐breeding periods (Wilson et al. 1999). Besides food resources, SRS also offers suitable shelter and cover from predators, as well as refuge from adverse weather conditions and farming practices. This was previously demonstrated for birds (Boatman et al. 2003; Vickery et al. 2009), European hares (Petrovan et al. 2013; Meichtry‐Stier et al. 2014; Mayer et al. 2018; Šálek et al. 2023), and small mammals (Briner et al. 2005; Šálek, Bažant, et al. 2022), including common hamsters (Fischer and Wagner 2016). For example, farming practices (like crop harvesting) can increase the risk of predation and mortality of juvenile European hares (Voigt and Siebert 2020; Cukor et al. 2024) and can destroy hamsters' burrow systems (La Haye et al. 2014). Therefore, SRS and other wildlife strips may represent highly valuable habitats that support their persistence in arable‐dominated farmlands (see also Fischer and Wagner 2016).

Our results also demonstrated that birds and European hares select SRS differently during the year, likely because of the seasonal changes in their demands. The quality of the SRS habitat also plays a key role due to seasonal farming operations, vegetation composition, and succession that affect the actual availability of essential food and cover resources (see also Marshall and Moonen 2002; Vickery et al. 2009; Dicks et al. 2013). More specifically, the SRS vegetation structure is highly dynamic throughout the year. During the autumn and early winter, SRS provide rich seed and cover resources. However, their suitability (and resource availability) for wildlife species gradually decreases in late winter (i.e., February) and then again in April when SRS are plowed and re‐sown (see Figure 2). Nevertheless, the subsequent natural succession of SRS vegetation in the spring and summer months creates dense, complex, and developed vegetation cover in early summer. For example, vegetation reaches ~50 cm in height in July (Boháč and Šálek, unpublished data), while the majority of the adjacent crop fields, especially those with cereals and rapeseed (i.e., the most common crops in our study area; see Section 2), are harvested and subsequently mechanically (soil cultivation) or chemically managed (using herbicide use for post‐harvest control of emergent weeds). This substantially reduces resource availability. Accordingly, we found that the abundance of farmland birds in SRS was significantly higher compared with control arable habitats from late summer to the end of winter (more precisely, in July and from November to March for all farmland birds, and from July to April for a subset of seed‐eating bird species). The abundance of European hares was significantly higher only from November to March. Moreover, our data have also shown that the largest differences in species diversity of farmland birds between the SRS and the control arable land were observed during late summer and autumn (from August to November). Conversely, the greatest differences in bird abundance were recorded in winter months (specifically, from December to February).

Our results, therefore, align with previous evidence showing that SRS provides increased plant food resources for birds in late autumn and winter when seeded plants and wild species offer peak seed availability (Henderson et al. 2007; Vickery et al. 2009; Dicks et al. 2013; Šálek, Bažant, et al. 2022). This may help birds (especially seed‐eating species) increase their overwinter survival, as various seed‐rich habitats are crucial foraging habitats for sedentary and wintering granivorous species (Robinson and Sutherland 1999; Robinson et al. 2004; Šálek, Havlíček, et al. 2015). In contrast to previous studies, we did not find a reduced abundance of farmland birds in SRS in late winter, when seed resources and vegetation cover can be largely depleted (cf. Henderson et al. 2007; Šálek, Bažant, et al. 2022). The importance of SRS for European hares during autumn and winter may be caused by a combination of high‐quality resting and fodder sources, when the high diversity of weeds and grasses provides valuable and preferable food resources for the species. Similarly, previous research showed that field margin strips provide improved food availability for European hares in the autumn and winter (Schai‐Braun et al. 2015), and preferable habitats with structurally rich vegetation for resting (Neumann et al. 2011; Petrovan et al. 2013; Pavliska et al. 2018; Šálek, Bažant, et al. 2022; Šálek et al. 2023).

Notably, our data also indicates that SRS may be indispensable for farmland bird species, not only throughout overwintering but also during the breeding, post‐breeding and migration periods. In particular, we documented the most profound differences in bird species diversity in SRS and controls from late summer to autumn (i.e., August to November). These findings provide compelling evidence that field margins support not only sedentary bird species but also a substantial number of migratory species that may use these food‐rich habitats for refueling during migration. In addition to plant food resources, SRS also provides high availability of invertebrates, a vital dietary component for many birds during those periods. Although the invertebrate abundance or species diversity in SRS is usually lower than in other types of field margins, such as uncropped field margins or sown wildflower strips (both directly oriented for supporting insect populations, see Vickery et al. 2009; Haaland et al. 2011), still they can offer highly valuable invertebrate foods for various bird species, including seed‐eaters, during the breeding and post‐breeding periods (see also Vickery et al. 2009).

Finally, the habitat context of study plots (i.e., SRS and controls situated within the crop field or along farmland hedges) did not affect the abundance and species diversity of farmland birds. However, seed‐eating bird diversity and abundance were higher in study plots situated along hedges, whereas the opposite pattern was found for the European hare. This is consistent with our previous study, which showed similar abundance and species diversity of farmland birds in SRS with diverse habitat contexts (field interiors, hedge, and forest; see Šálek, Bažant, et al. 2022). This suggests that individual bird species respond differently to the spatial location of SRS within the landscape, probably reflecting their species‐specific differences in habitat selection (Šálek, Bažant, et al. 2022). However, in contrast to the previous study, which focused only on the winter period, our current research shows that the implementation of SRS in a variety of locations/contexts can support higher abundance and species diversity of farmland wildlife species throughout the year. Higher species diversity and abundance of seed‐eating birds in study plots situated along hedges (especially during the post‐breeding period) likely reflect the benefits of structurally diverse shrub and tree vegetation of hedges (Šálek, Kalinová, and Reif 2022). These hedgerows provide extra nesting opportunities, safe hides, and food resources (e.g., berries and fruits), and thus support a higher number and diversity of seed‐eating bird species (Hinsley and Bellamy 2000). A lower abundance of European hares in plots along hedges, which is consistent with previous studies and confirms the hares' habitat affinity to open agricultural landscapes (Mayer et al. 2018; Pavliska et al. 2018)—among other things, to avoid predation risk by mammalian predators, whose foraging activity mostly focuses on different types of habitat boundaries, including farmland hedges (Šálek et al. 2010, 2014; Červinka et al. 2011, 2013).

### 4.1 Limitations of the Study

Although our study demonstrates clear ecological benefits of SRS for farmland birds and two representatives of mammals (European hares and common hamsters), several limitations should be acknowledged. In particular, while the number of replicates per treatment may appear limited, our intensive sampling effort (10 study plots × 12 months = 120 surveys) generated a substantial dataset (i.e., ~4000 individuals of 46 farmland bird species). Similarly, our data were collected over a single annual cycle within the Central European cropland‐dominated farming systems, which may limit direct generalization to other climatic regions, contrasting farmlands, or multi‐year population dynamics. However, the ability to detect significant effects across multiple taxa/species and seasons—particularly during critical autumn/winter periods—suggests that our sampling design provided adequate statistical power to reveal meaningful biological patterns (see also Van Wilgenburg et al. 2024). The consistency of our findings across months and parameters (bird abundance/diversity, mammal abundance) indicates that we identified robust, reproducible ecological relationships rather than artifacts. This temporal consistency also aligns with previous local or regional studies (Boatman et al. 2003; Šálek, Bažant, et al. 2022) and supports the conclusion that SRS might bring the highest ecological benefits to farmland vertebrates during the late autumn and winter due to enhanced resource availability in SRS. However, while our single‐year dataset demonstrated clear seasonal patterns, we recommend further detailed multi‐year investigations of the SRS conservation potential and long‐term population impact for various farmland species. For example, SRS selection by different bird and mammal species can change interannually depending on meteorological conditions within a particular year (e.g., dry vs. wet year, harsh vs. mild winter), affecting habitat quality (i.e., vegetation structure and, therefore, the seed and cover resource availability) of the SRS or the species distribution/pool within the landscape. Similarly, future research should focus on a higher diversity of mammalian species with various life history traits and expand the geographic scope beyond Central European contexts to evaluate the generalizability of our findings across different wildlife species and diverse agricultural systems.

## 5 Conclusions and Conservation Implications

This study enhances our understanding of the SRS conservation potential across the full annual cycle of farmland birds and European hares. We conclude that implementing SRS can substantially contribute to year‐round conservation of avian and selected mammalian species and potentially help to increase their population numbers in arable‐dominated farmlands (see also Buckingham et al. 1999; Siriwardena et al. 2000). Notably, promoting SRS may benefit studied wildlife species not only in the autumn and winter, when dense and structurally complex vegetation in SRS offers plentiful nutrient‐rich seeds and cover, but also in the summer when SRS provide invertebrates for various farmland bird species. Increasing availability of high‐quality food resources in arable habitats throughout the year is critical due to long‐term and large‐scale decreases in the representation of other seed‐rich habitats, such as stubble fields and set‐asides (Robinson and Sutherland 2002).

Despite SRS's benefits, there is still considerable room for improving the conservation potential and food availability of SRS throughout the year. Potential enhancements include adding seed mixtures to increase seed availability during late winter (Boatman et al. 2003; Šálek, Bažant, et al. 2022). Additionally, implementing spatio‐temporal management strategies, such as selective cutting, could increase accessibility to invertebrate species in summer (Perkins et al. 2002; Douglas et al. 2009). Moreover, a combination of alternative types of field margins with contrasting management regimes (e.g., uncropped field margins with a high diversity of perennial flowers), the timing of cultivation (spring vs. autumn cultivation), habitat context, vegetation composition and structure, may provide desired ecological advantages, such as higher abundance and diversity of seed resources in the winter and invertebrates in the summer or both (see also Schütz et al. 2022). Finally, despite their ecological benefits in supporting farmland biodiversity and ecosystem services (Dicks et al. 2013), their contribution to the esthetics of farmland (a quality valued by the general public; De Snoo et al. 2023), and simple establishment and management (Haaland et al. 2011), SRS and various field margins are implemented on a small fraction of arable land (Pe'er et al. 2017; Šálek, Bažant, et al. 2022). Thus, they may rarely halt farmland biodiversity loss at the landscape scale. Therefore, improving AEC‐based funding for SRS and other food‐rich habitats on arable land (i.e., set‐asides, fallow land, organic farming, and other types of field margins), along with better promotion among farmers and reducing administrative burdens (Pe'er et al. 2017), might facilitate their implementation and thereby optimize their ecosystem services.

## Author Contributions

**Martin Šálek:** conceptualization (lead), data curation (equal), formal analysis (supporting), funding acquisition (equal), project administration (equal), resources (equal), supervision (lead), writing – original draft (lead). **Ondřej Boháč:** investigation (lead), methodology (equal), writing – review and editing (supporting). **Jan Cukor:** conceptualization (equal), funding acquisition (lead), methodology (equal), resources (equal), writing – review and editing (equal). **Peter Samaš:** data curation (lead), formal analysis (lead), software (lead), validation (equal), visualization (lead), writing – original draft (lead).

## Conflicts of Interest

The authors declare no conflicts of interest.

## Supporting information


**Data S1:** ece372492‐sup‐0001‐DataS1.csv.

**Data S2:** ece372492‐sup‐0002‐DataS2.R.

**Tables S1–S5:** ece372492‐sup‐0003‐TablesS1‐S5.docx.

## Data Availability

The data, Supporting Information tables, and R code supporting the results of this study are openly available in the Dryad Digital Repository at https://datadryad.org/share/10.5061/dryad.m0cfxpph1.
