# Supplementary material for: Year‐Round Evaluation of the Conservation Potential of Seed‐Rich Field Margins Under Agri‐Environmental Schemes for Farmland Birds, European Hares, and Common Hamsters
Source: Ecol Evol. 2025 Nov 18;15(11):e72492. doi: 10.1002/ece3.72492 (PMC12626644; doi:10.1002/ece3.72492)
Supplement: Supplementary file 3 — Tables S1–S5: ece372492‐sup‐0003‐TablesS1‐S5.docx. [file ECE3-15-e72492-s003.docx]

**Supplementary material**

**Table S1.** The complete enumeration of mandatory and optional plant species utilized for the establishment of SRS and their minimum quantity in the mixture (kg/ha).

|  |  |  |
| --- | --- | --- |
|  |  | Minimum amount in the mixture (kg/ha) |
| **Mandatory species** |  |  |
| Spring cereals | *Avena sativa*, *Triticum aestivum*, or *Hordeum vulgare* | 65 |
| Millet | *Panicum miliaceum* | 15 |
| Wild cabbage | *Brassica oleracea* | 0.8 |
| Buckwheat | *Fagopyrum esculentum* | 15 |
|  |  |  |
| **Optional species** |  |  |
| Common sunflower | *Helianthus annuus* | 2.5 |
| Canary grass | *Phalaris canariensis* | 5 |
| Lacy phacelia | *Phacelia tanacetifolia* | 5 |
| Blue curls | *Phacelia congesta* | 5 |
| Flax | *Linum usitatissimum* | 20 |
| Legumes | *Pisum sativum*, *Vicia faba*, or *Vicia sativa* | 30 |
| White lupin | *Lupinus albus* | 5 |
| Sorghum | *Sorghum bicolor* | 3 |
| Foxtail millet | *Setaria italica* | 4 |

**Table S2**. A list of farmland species recorded in SRS and corresponding control transects, their diet, sum of individuals, mean abundance per point and SD for both habitats (SRS and controls) types. For each species, mean abundance and SD were calculated only across points where the species was observed (i.e., presence data), excluding absences.

|  |  |  |  | **SRS** |  |  | **Control** |  |  |
| --- | --- | --- | --- | --- | --- | --- | --- | --- | --- |
| **English name** | **Species** | **Group** | **Diet** | **Sum** | **Mean** | **SD** | **Sum** | **Mean** | **SD** |
| Marsh Warbler | *Acrocephalus palustris* | birds | animals | 4 | 1.33 | 0.58 | 0 | NA | NA |
| Eurasian Skylark | *Alauda arvensis* | birds | seeds | 65 | 2.41 | 1.62 | 45 | 1.55 | 1.09 |
| Meadow Pipit | *Anthus pratensis* | birds | animals | 14 | 3.50 | 4.36 | 3 | 1.50 | 0.71 |
| Short-eared Owl | *Asio flammeus* | birds | vertebrates | 2 | 1.00 | 0.00 | 0 | NA | NA |
| Eurasian Buzzard | *Buteo buteo* | birds | vertebrates | 92 | 1.84 | 1.18 | 68 | 1.84 | 1.21 |
| Rough-legged Buzzard | *Buteo lagopus* | birds | vertebrates | 10 | 1.43 | 0.54 | 6 | 1.50 | 0.58 |
| European Goldfinch | *Carduelis carduelis* | birds | seeds | 128 | 5.33 | 5.90 | 19 | 1.58 | 0.67 |
| Redpoll | *Carduelis flammea* | birds | seeds | 5 | 5.00 | NA | 0 | NA | NA |
| European Greenfinch | *Chloris chloris* | birds | seeds | 7 | 1.40 | 0.89 | 10 | 5.00 | 5.66 |
| White Stork | *Ciconia ciconia* | birds | vertebrates | 0 | NA | NA | 5 | 2.50 | 2.12 |
| Western Marsh-harrier | *Circus aeruginosus* | birds | vertebrates | 15 | 2.14 | 0.90 | 18 | 2.57 | 1.72 |
| Hen Harrier | *Circus cyaneus* | birds | vertebrates | 2 | 1.00 | 0.00 | 0 | NA | NA |
| Pallid Harrier | *Circus macrourus* | birds | vertebrates | 2 | 1.00 | 0.00 | 0 | NA | NA |
| Montagu's Harrier | *Circus pygargus* | birds | vertebrates | 0 | NA | NA | 3 | 1.50 | 0.71 |
| Common Raven | *Corvus corax* | birds | omnivore | 26 | 1.86 | 1.29 | 27 | 2.08 | 2.02 |
| Carrion Crow | *Corvus corone* | birds | omnivore | 79 | 2.93 | 2.90 | 57 | 3.17 | 3.50 |
| Eurasian Jackdaw | *Corvus monedula* | birds | omnivore | 12 | 4.00 | 3.46 | 24 | 4.80 | 2.78 |
| Yellowhammer | *Emberiza citrinella* | birds | seeds | 234 | 9.00 | 11.01 | 84 | 3.11 | 2.75 |
| Reed Bunting | *Emberiza schoeniclus* | birds | seeds | 51 | 3.64 | 2.79 | 1 | 1.00 | NA |
| Merlin | *Falco columbarius* | birds | vertebrates | 1 | 1.00 | NA | 0 | NA | NA |
| Common Kestrel | *Falco tinnunculus* | birds | vertebrates | 50 | 1.47 | 0.83 | 35 | 1.40 | 0.76 |
| Red-footed Falcon | *Falco vespertinus* | birds | vertebrates | 0 | NA | NA | 2 | 2.00 | NA |
| Barn Swallow | *Hirundo rustica* | birds | animals | 8 | 2.00 | 0.82 | 0 | NA | NA |
| Red-backed Shrike | *Lanius collurio* | birds | animals | 7 | 1.17 | 0.41 | 2 | 1.00 | 0.00 |
| Great Grey Shrike | *Lanius excubitor* | birds | vertebrates | 0 | NA | NA | 2 | 1.00 | 0.00 |
| Common Linnet | *Linaria cannabina* | birds | plants | 620 | 36.47 | 94.61 | 7 | 1.75 | 0.96 |
| Black Kite | *Milvus migrans* | birds | vertebrates | 1 | 1.00 | NA | 1 | 1.00 | NA |
| Red Kite | *Milvus milvus* | birds | vertebrates | 0 | NA | NA | 1 | 1.00 | NA |
| White Wagtail | *Motacilla alba* | birds | animals | 7 | 1.75 | 0.96 | 3 | 3.00 | NA |
| Western Yellow Wagtail | *Motacilla flava* | birds | animals | 3 | 1.50 | 0.71 | 4 | 1.00 | 0.00 |
| Northern Wheatear | *Oenanthe oenanthe* | birds | animals | 1 | 1.00 | NA | 0 | NA | NA |
| Eurasian Tree Sparrow | *Passer montanus* | birds | seeds | 633 | 12.17 | 19.38 | 137 | 9.79 | 7.61 |
| Grey Partridge | *Perdix perdix* | birds | seeds | 15 | 5.00 | 3.46 | 8 | 2.67 | 1.16 |
| Common Pheasant | *Phasianus colchicus* | birds | seeds | 24 | 2.40 | 2.17 | 5 | 1.00 | 0.00 |
| Eurasian Magpie | *Pica pica* | birds | omnivore | 46 | 2.71 | 3.26 | 41 | 1.95 | 0.92 |
| Common Stonechat | *Saxicola torquata* | birds | animals | 2 | 1.00 | 0.00 | 0 | NA | NA |
| European Serin | *Serinus serinus* | birds | seeds | 2 | 2.00 | NA | 1 | 1.00 | NA |
| Eurasian Collared-dove | *Streptopelia decaocto* | birds | seeds | 0 | NA | NA | 2 | 2.00 | NA |
| European Turtle-dove | *Streptopelia turtur* | birds | seeds | 3 | 1.50 | 0.71 | 0 | NA | NA |
| Common Starling | *Sturnus vulgaris* | birds | animals | 121 | 9.31 | 18.92 | 20 | 3.33 | 1.75 |
| Common Whitethroat | *Sylvia communis* | birds | animals | 12 | 2.00 | 0.00 | 5 | 1.00 | 0.00 |
| Redwing | *Turdus iliacus* | birds | seeds | 2 | 2.00 | NA | 0 | NA | NA |
| Fieldfare | *Turdus pilaris* | birds | seeds | 164 | 41.00 | 66.25 | 28 | 4.00 | 4.40 |
| Northern Lapwing | *Vanellus vanellus* | birds | animals | 9 | 2.25 | 1.26 | 2 | 1.00 | 0.00 |
| Common Hamster | *Cricetus cricetus* | mammals |  | 75 | 4.69 | 11.01 | 5 | 1.25 | 0.50 |
| European Hare | *Lepus europaeus* | mammals |  | 268 | 4.70 | 4.86 | 73 | 1.92 | 1.17 |
|  |  |  |  |  |  |  |  |  |  |

**Table S3.** Summary statistics of Generalized Additive Models (GAMs) testing ecological predictors of abundance for farmland birds (left columns), seed-eating birds (middle columns), and European hares (right columns). For each term, the statistical significance is given as the z-value (parametric terms) or χ² statistic (smooth terms), with corresponding effective degrees of freedom (edf), reference degrees of freedom (ref.df), and p-value (P).

|  |  | Farmland birds | | | | Seed-eating birds | | | | European hares | | | |
| --- | --- | --- | --- | --- | --- | --- | --- | --- | --- | --- | --- | --- | --- |
| Term type | Variable name | z/χ² value | edf | ref.df | P | z/χ² value | edf | ref.df | P | z/χ² value | edf | ref.df | P |
| Parametric | Intercept | −27.61 | – | – | <0.001 | −16.41 | – | – | <0.001 | −22.90 | – | – | <0.001 |
| Parametric | Habitat[SRS] | 7.73 | – | – | <0.001 | 8.08 | – | – | <0.001 | 3.86 | – | – | <0.001 |
| Parametric | Context[hedge] | 0.21 | – | – | 0.83 | 2.87 | – | – | 0.004 | −2.29 | – | – | 0.02 |
| Smooth | Month*control | 11.41 | 2.60 | 8 | 0.008 | 18.97 | 2.48 | 8 | <0.001 | 5.41 | 1.81 | 8 | 0.03 |
| Smooth | Month*SRS | 74.66 | 3.64 | 8 | <0.001 | 101.96 | 4.14 | 8 | <0.001 | 45.56 | 3.68 | 8 | <0.001 |
| Smooth | Species | 667.71 | 39.77 | 43 | <0.001 | 275.52 | 13.32 | 14 | <0.001 | 15.66 | – | – | – |
| Smooth | Plot ID | 54.05 | 6.97 | 8 | <0.001 | 36.93 | 6.54 | 8 | <0.001 | −22.90 | 5.40 | 8 | <0.001 |

**Table S4.** Summary statistics of Generalized Additive Models (GAMs) testing ecological predictors of alpha diversity (Shannon index) for farmland birds (left columns) and seed-eating birds (right columns). For each term, the statistical significance is given as the z-value (parametric terms) or χ² statistic (smooth terms), with corresponding effective degrees of freedom (edf), reference degrees of freedom (ref.df), and p-value (P).

|  |  | Farmland birds | | | | Seed-eating birds | | | |
| --- | --- | --- | --- | --- | --- | --- | --- | --- | --- |
| Term type | Variable name | z/χ² value | edf | ref.df | P | z/χ² value | edf | ref.df | P |
| Parametric | Intercept | −48.01 | – | – | <0.001 | −34.15 | – | – | <0.001 |
| Parametric | Habitat[SRS] | 4.47 | – | – | <0.001 | 3.42 | – | – | 0.001 |
| Parametric | Context[hedge] | 0.17 | – | – | 0.87 | 4.19 | – | – | <0.001 |
| Smooth | Month*control | 3.45 | 2.53 | 8 | <0.001 | 2.07 | 2.22 | 8 | <0.001 |
| Smooth | Month*SRS | 1.59 | 2.84 | 8 | 0.002 | 0.66 | 2.48 | 8 | 0.08 |
| Smooth | Plot ID | 5.69 | 6.36 | 8 | <0.001 | 0.42 | 2.11 | 8 | 0.14 |

**Table S5.** Summary statistics of Generalized Additive Models (GAMs) testing ecological predictors of abundance for common hamster. For each term, the statistical significance is given as the z-value (parametric terms) or χ² statistic (smooth terms), with corresponding effective degrees of freedom (edf), reference degrees of freedom (ref.df), and p-value (P).

| Term type | Variable name | z/χ² value | edf | ref.df | P |
| --- | --- | --- | --- | --- | --- |
| Parametric | Intercept | −7.08 | – | – | <0.001 |
| Parametric | Habitat[SRS] | 3.70 | – | – | <0.001 |
| Parametric | Context[hedge] | −1.72 | – | – | 0.09 |
| Smooth | Plot ID | 14.9 | 5.60 | 8 | 0.009 |
